# Lipocalin-2: a therapeutic target to overcome neurodegenerative diseases by regulating reactive astrogliosis

**DOI:** 10.1038/s12276-023-01098-7

**Published:** 2023-10-02

**Authors:** Byung-Kwon Jung, Kwon-Yul Ryu

**Affiliations:** Department of Life Science, University of Seoul, Seoul, 02504 Republic of Korea

**Keywords:** Macroautophagy, Proteasome, Cell death in the nervous system, Stress signalling, Astrocyte

## Abstract

Glial cell activation precedes neuronal cell death during brain aging and the progression of neurodegenerative diseases. Under neuroinflammatory stress conditions, lipocalin-2 (LCN2), also known as neutrophil gelatinase-associated lipocalin or 24p3, is produced and secreted by activated microglia and reactive astrocytes. *Lcn2* expression levels are known to be increased in various cells, including reactive astrocytes, through the activation of the NF-κB signaling pathway. In the central nervous system, as LCN2 exerts neurotoxicity when secreted from reactive astrocytes, many researchers have attempted to identify various strategies to inhibit LCN2 production, secretion, and function to minimize neuroinflammation and neuronal cell death. These strategies include regulation at the transcriptional, posttranscriptional, and posttranslational levels, as well as blocking its functions using neutralizing antibodies or antagonists of its receptor. The suppression of NF-κB signaling is a strategy to inhibit LCN2 production, but it may also affect other cellular activities, raising questions about its effectiveness and feasibility. Recently, LCN2 was found to be a target of the autophagy‒lysosome pathway. Therefore, autophagy activation may be a promising therapeutic strategy to reduce the levels of secreted LCN2 and overcome neurodegenerative diseases. In this review, we focused on research progress on astrocyte-derived LCN2 in the central nervous system.

## Astrocyte-derived LCN2: a new target in neurodegenerative diseases

Astrocyte-secreted protein Lipocalin-2 (LCN2) may hold the key to preventing neurodegenerative diseases, according to a new study. Researchers found that LCN2, a neurotoxin associated with neuroinflammation and neurotoxicity, is secreted by activated astrocytes in the brain. By reducing LCN2 levels in these astrocytes, it may be possible to convert them from a neurotoxic state to a neuroprotective one, potentially preventing neuronal cell death and the progression of neurodegenerative diseases. This novel approach could provide an alternative to current therapies that focus solely on neurons and offer new hope for treating conditions such as Alzheimer’s and Parkinson’s disease.

## Introduction

In the central nervous system (CNS), astrocytes and microglia perform diverse cellular functions to maintain brain homeostasis^1–4^. Astrocyte activation, or reactive astrogliosis, is closely associated with the progression of various neurodegenerative diseases^5–7^. During the progression of Alzheimer’s disease, neuroinflammation caused by reactive astrogliosis occurs before the onset of dementia symptoms and neurodegeneration^8^. Mouse models with similar phenotypes, such as polyubiquitin gene *Ubb* knockout (KO) mice, in which neuronal cell death occurs following astrocyte activation in the hypothalamic region of the brain, have been developed to study this phenomenon^9–11^. According to the traditional dichotomous concept, activated astrocytes, also known as reactive astrocytes, can be classified into two subtypes: A1 and A2. These subtypes exhibit different transcriptome profiles, with the A1 subtype associated with neuroinflammation and neurotoxicity and the A2 subtype exerting neuroprotective actions^5,8,12^. Lipocalin-2 (LCN2) is a neurotoxin and marker protein that reflects or even determines the state and phenotype of activated astrocytes^13,14^. LCN2 levels are higher in the A1 subtype than in the A2 subtype. The expression level of *Lcn2* is increased through the activation of the nuclear factor kappa B (NF-κB) signaling pathway under inflammatory stress conditions^15–17^. Although some aspects of the transcriptional regulation of *Lcn2* in the brain and other tissues are known and its association with matrix metalloproteinase-9 (MMP-9) is well-established, the impact of complex formation on LCN2 function and its regulation at the posttranslational level has only recently begun to be understood^18–22^.

In models of brain injury or stroke, the function of LCN2 appears to be complex, and it can exhibit neurotoxic or neuroprotective effects, depending on the context. Nevertheless, significant efforts have been made to develop LCN2 as a therapeutic target^23^. Numerous strategies have been proposed to overcome neurodegeneration and to mitigate the neurotoxic effects of LCN2. These strategies include inhibiting *Lcn2* expression, blocking the function of secreted LCN2 and its receptor 24p3R, and suppressing LCN2 receptor signaling^24^. However, these approaches have certain limitations. To inhibit LCN2 function, one possible approach is to downregulate *Lcn2* expression at the transcriptional level by inhibiting NF-κB signaling^22^. However, given the redundant nature of signal transduction pathways, inhibiting one signaling pathway may activate another, making it uncertain whether an effective and sustained reduction in *Lcn2* expression is achievable. Furthermore, inhibition of signal transduction pathways may be reversible if the inhibitor is no longer present. In addition, inhibition at the protein level by inhibiting LCN2 secretion or blocking its binding to the LCN2 receptor 24p3R may also be reversible. In contrast, promoting the degradation of LCN2 protein may result in the irreversible inhibition of LCN2 function. This approach holds promise for achieving sustained suppression of LCN2 activity^22^.

In this review, we cautiously propose that future research should focus on indirectly targeting astrocytes, rather than directly targeting neurons, to overcome neurodegeneration. This strategy is based on the idea that the activated state of astrocytes can be altered to induce neuroprotective effects and prevent neuronal cell death. Reducing LCN2 levels in activated astrocytes is expected to modify their transcriptome profiles, leading to a decrease in A1 marker expression levels and an increase in A2 marker expression levels^22^. In neurodegenerative diseases, neurodegeneration typically occurs after astrocyte activation. The level of LCN2 is highest during astrocyte activation and the early stages of neurodegeneration, such as in the mild cognitive impairment stage of Alzheimer’s disease^25^. Therefore, measuring the level of LCN2 secreted by activated astrocytes, potentially in the blood plasma and urine, may serve as an early indicator of and diagnostic tool for neurodegenerative diseases. Furthermore, manipulating the activated state of astrocytes by reducing LCN2 levels is a potential therapeutic strategy for overcoming neurodegenerative diseases.

## Glia in the central nervous system

Since their first identification in the 19th century by pioneers in the field, including Rudolf Virchow, glial cells (with “glia” meaning “glue” in Greek) have been recognized as nonneuronal cells that account for ~50% of the total cells in the adult human brain. They have been shown to play multifunctional roles, from early nervous system development to late-onset neuropathological processes^26–28^. Glial cells in the CNS are subclassified into astrocytes, microglia, and oligodendrocytes based on their molecular characteristics, functions, and morphologies^29^. Oligodendrocytes differentiate from neural/glial antigen 2 (NG2) proteoglycan-positive progenitors during brain development and perform unique functions among glial cells. Their primary role is to form specialized cellular compartments, known as myelin sheaths, around the neurons’ axons^30,31^. Unlike other glial cells, microglia originate from embryonic hematopoietic precursors. They exhibit phagocytic activity similar to that of macrophages and orchestrate immune responses within the CNS, participating in the maintenance of brain homeostasis^32^. Finally, astrocytes, the most abundant glial cells in the CNS, originate from neural progenitors and undergo differentiation and maturation primarily after birth^2,33^. Astrocytes have diverse functions that are critical for proper brain function. They provide structural support, regulate extracellular ion balance, modulate neurotransmitter activity, support metabolic processes, participate in blood‒brain barrier (BBB) regulation, and perform other essential tasks to support neuronal health and function.

## Astrocyte functions in normal physiology

Astrocytes can be divided into two major classes based on their morphology and location: protoplasmic astrocytes, primarily found in the gray matter, and fibrous astrocytes, located in the white matter^34,35^. Both subclasses of astrocytes have extensive processes that make contact with blood vessels in the CNS, allowing their participation in the formation and regulation of the BBB^36,37^. However, the ability of astrocytes to induce the BBB in vivo remains controversial. Their interaction with the BBB and their secretion of various molecules, such as prostaglandins and nitric oxide (NO), regulate blood flow when the activity of neighboring neurons changes^38–41^. Moreover, astrocytic processes completely envelop the synapses and nodes of Ranvier in the CNS. These processes involve a variety of channels and transporters, enabling astrocytes to monitor essential elements, such as fluids, ions, and neurotransmitters. They regulate these components to maintain a healthy synaptic microenvironment^42,43^. In addition to their role in regulating synaptic homeostasis, astrocytes act as “tuners” on a larger scale during CNS development. They promote synaptogenesis and eliminate synapses by pruning^35,44,45^. These diverse functions of astrocytes in a healthy CNS are meticulously carried out throughout life. However, when something goes wrong, astrocytes recognize the disturbance and become more specialized to correct potentially harmful perturbations, a phenomenon known as “reactive astrogliosis”.

## Reactive astrogliosis: beyond conventional glial scar concepts and markers

Neuroscientists have reported a growing body of evidence regarding distinctive scarring phenomena in the CNS, such as “filling destroyed space and building a wall”, which occurs in response to severe damage or neuroinflammatory insults^46^. One well-known cause of glial scarring is traumatic brain injury, a neurodegenerative and noncongenital insult to the CNS. Glial scarring has been observed in both experimental animal models and patient cases^47,48^. Other triggers of glial scarring include stroke, ischemia, autoimmune responses, and even severe progression of neurodegenerative diseases^49–52^. In most of the cases mentioned above, astrocytes undergo morphological changes characterized by intensive overlapping processes in local lesions. These molecular and cellular alterations, known as “reactive astrogliosis”, are considered a major requirement for the formation of glial scars. Initially, the prevailing concept of glial scarring and reactive astrogliosis was that glial scarring inhibited axonal regeneration, thus obstructing tissue regeneration after CNS injury. Based on the observed morphology in conventional immunohistology, glial scars with reactive astrogliosis serve as barriers to prevent the infiltration of local immune cells and infectious factors into CNS lesions and protect nondamaged neurons from nearby intense inflammatory responses^53^. However, in addition to these neuroprotective functions, it remains controversial whether glial scar formation itself contributes to neuronal loss and degeneration, particularly in neurodegenerative diseases, such as Alzheimer’s and Parkinson’s diseases^54^. Furthermore, because glial scar formation is typically observed in the later stages of animal models or patient cases, it is essential to thoroughly investigate mild and moderate reactive astrogliosis without glial scar formation. Such investigations may aid in the control and reduction of extensive neuronal loss, which may alleviate neurodegenerative symptoms.

Glial fibrillary acid protein (GFAP), a component of intermediate filaments in CNS cells, has been widely used as a molecular marker of astrocytes under both normal and pathological conditions^55^. However, it is important to not overlook the inherent characteristics of GFAP. Numerous astrocytes in the healthy CNS cannot be detected by conventional GFAP staining because GFAP was initially discovered in isolated plaques from multiple sclerosis patients^56^. Several studies have indicated that GFAP expression is not related to the normal physiology of astrocytes in the healthy CNS. However, in pathophysiological conditions such as glial scar formation following CNS injury, GFAP and its upregulation are necessary. Therefore, GFAP expression levels are more useful and reliable for detecting reactive astrogliosis in response to CNS insults than for identifying normal astrocytes under healthy conditions.

## Brain aging, neurodegeneration, and reactive astrogliosis

Glial cells, including astrocytes, microglia, and oligodendrocytes, account for 50% of all brain cells. Astrocytes are the most abundant glial cells, comprising 10–20% of all brain cells^57^, while microglia, known as brain macrophages, as they support immune surveillance, account for 5–10%^3,4^. During brain aging, glial cells are the first cells to undergo alterations, and these changes are closely related to neurodegenerative diseases. With aging, myelin fragmentation occurs, forming insoluble lysosomal inclusions in microglia, which deteriorate their immune function^58^. Astrocytes perform various functions, including supporting neurons and controlling their growth, participating in synapse formation, regulating synaptic plasticity, and contributing to the regulation of the BBB^59^. As brain aging progresses, astrocytes are generally activated, leading to increased production of complement proteins. This activation pattern is also observed in the early stages of neurodegenerative diseases^60^.

Unlike previous statistical studies that showed a correlation between age and the risk of neurodegenerative diseases, neurobiologists have discussed whether aging and its effects are the true causes of these diseases^61,62^. With significant advancements in our understanding of the characteristics of aging and CNS neurodegeneration, it has become more comprehensible how aging is involved in neurodegenerative diseases and their pathophysiological progression^63,64^. All identified hallmarks of aging are closely related to neurodegenerative diseases, including Alzheimer’s and Parkinson’s diseases, in both sporadic and familial cases^65^. Two of these hallmarks, the loss of proteostasis and altered intercellular communication, are particularly noteworthy, as they contribute to both neurodegeneration and reactive astrogliosis under these pathological conditions. In Alzheimer’s and Parkinson’s diseases, the most common neurodegenerative diseases characterized by proteinopathies, specific proteins aggregate in the cytoplasm or extracellular space of neurons. During the severe progression of Alzheimer’s disease, amyloid-β and phosphorylated tau form insoluble inclusion bodies known as amyloid plaques and neurofibrillary tangles, respectively. In Parkinson’s disease, there is a significant correlation between disease incidence and the presence of specific protein aggregates called Lewy bodies, which are mainly composed of α-synuclein. In this context, reinforcing the aging-induced loss of proteostasis in both neurons and astrocytes is a therapeutic challenge. Furthermore, altered intercellular communication has been implicated in these diseases, particularly between neurons and astrocytes. Reactive astrocytes observed in lesions gradually lose their neuroprotective and neuromanagerial functions and gain the ability to secrete inflammatory cytokines and complement proteins. Therefore, the modulation of reactive astrocytes under pathophysiological conditions, primarily induced by the activation of proinflammatory signaling, can be achieved by targeting the essential steps before their activation or during their maintenance.

Neurotoxins such as LCN2, secreted by activated astrocytes, promote neuronal cell death^66^. Therefore, astrocyte activation precedes neurodegeneration. In activated astrocytes, functions such as neuronal support are weakened, and immune and inflammatory responses are activated. The number of astrocytes does not generally increase as they become active; however, their morphology and function change along with alterations in their gene expression or transcriptome profiles. Various outcomes of astrocyte activation have been reported depending on the type of neurodegenerative disease and affected brain region^5^. However, it is not well understood how the function and activation of astrocytes change during the onset and progression of neurodegenerative diseases or how they contribute to neuronal cell death. Therefore, regulation of the activated state of astrocytes may serve as a breakthrough therapeutic strategy for inhibiting the progression of neurodegenerative diseases.

## Two subtypes of reactive astrocytes based on the traditional dichotomous concept

**Fig. 1:**
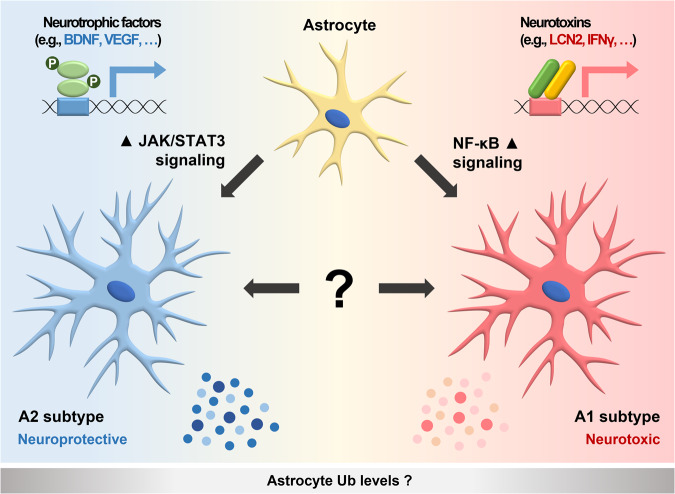
Two subtypes of activated astrocytes. Astrocytes have the ability to be activated into the A1 subtype, which is characterized by elevated expression levels of proinflammatory cytokines. These reactive astrocytes, through activation of the nuclear factor kappa B (NF-κB) signaling pathway, secrete neurotoxins that can contribute to neurodegeneration. Astrocytes can also be activated into the A2 subtype, which exhibits high levels of anti-inflammatory cytokines. These reactive astrocytes, through activation of the Janus kinase/signal transducer and activator of transcription 3 (JAK/STAT3) signaling pathway, secrete neurotrophic factors that exert neuroprotective effects. Whether the levels of ubiquitin (Ub) in astrocytes have an impact on their activation status remains unknown.

Astrocytes exhibit different gene expression profiles, depending on their activation status. Because changes in gene expression are a continuous process, it can be inferred that reactive astrocytes exist in numerous different states^67^. Additionally, the status of reactive astrocytes should be considered in a context-dependent manner because it can be influenced by the surrounding microenvironment^68^. Despite these variations, reactive astrocytes with distinct gene expression profiles exist^69^. In this review, we propose defining reactive astrocytes as the A1 subtype if they exhibit high levels of A1 marker expression and as the A2 subtype if they display high levels of A2 marker expression (Fig. 1). The A1 subtype is characterized by high expression levels of proinflammatory cytokines and *Lcn2*, while the A2 subtype is associated with high expression levels of anti-inflammatory cytokines and low expression levels of *Lcn2*. The A1 subtype, also known as inflammatory or classical reactive astrocytes, emerges in response to inflammatory stress. Conversely, the A2 subtype, known as ischemic or alternative reactive astrocytes, appears after ischemic brain injury or neuronal damage^70^. Although the A1 subtype arises after inflammatory stress and the A2 subtype emerges after ischemic brain injury, both subtypes may coexist^71^. Specific subtypes of reactive astrocytes are also influenced by changes in the surrounding microenvironment and cell signaling^8,69^. Notably, we do not support the notion that there are only two distinct subtypes of reactive astrocytes. Instead, we suggest that each reactive astrocyte has a unique transcriptome profile^68^. Similarly, activated microglia cannot simply be categorized into M1 and M2 subtypes, as each activated microglial cell exhibits a unique transcriptome profile^72^.

Based on transcriptome analysis, the activation of NF-κB signaling has been observed in the A1 subtype of reactive astrocytes. This activation leads to the secretion of proinflammatory cytokines, including tumor necrosis factor alpha (TNFα), interferon-gamma (IFNγ), and interleukin-1 beta (IL-1β), as well as neurotoxins, such as LCN2. Consequently, neuroinflammation and neuronal cell death occur^13,66,73^ (Fig. 1). In contrast, in the A2 subtype, Janus kinase/signal transducer and activator of transcription 3 (JAK/STAT3) signaling is activated, promoting the secretion of anti-inflammatory cytokines, such as tumor growth factor beta (TGFβ) and interleukin-10 (IL-10), as well as neurotrophic factors, such as brain-derived neurotrophic factor (BDNF) and vascular endothelial growth factor (VEGF). This leads to neuroprotection (Fig. 1). Both subtypes coexist in the brain; however, as neurodegenerative diseases progress, the prevalence of the A1 subtype increases. Similar to the M1-M2 conversion observed in microglia, which are classified as neurotoxic M1 and neuroprotective M2 subtypes^74,75^, there is a possibility of converting reactive astrocytes from the A1 subtype to the A2 subtype^76^. Interferon regulatory factor 3 (IRF3) has been shown to suppress proinflammatory cytokine gene expression through miRNA regulation, thereby converting reactive astrocyte subtypes from the proinflammatory A1 subtype to the anti-inflammatory A2 subtype, which protects neurons from apoptosis^76^. Therefore, inducing the subtype conversion of reactive astrocytes to alter their effects on surrounding neurons, rather than inhibiting astrocyte activation, may serve as a promising therapeutic strategy for overcoming neurodegenerative diseases.

## The role of LCN2 in various organs and in the CNS

**Fig. 2:**
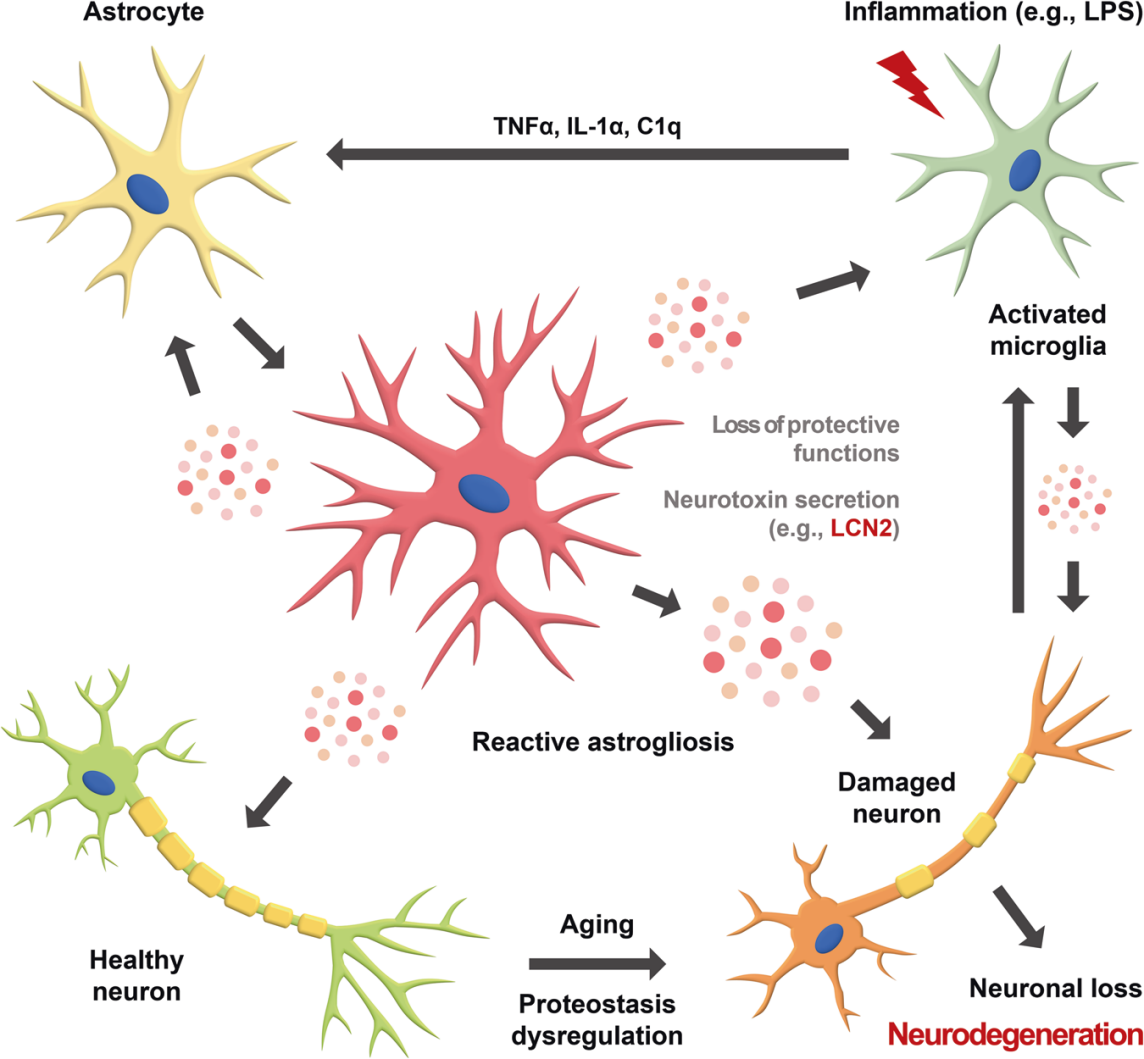
Neurotoxicity induction model of activated astrocytes. Under inflammatory stress induced by the administration of lipopolysaccharide (LPS), microglia become activated and secrete proinflammatory cytokines, such as TNFα, IL-1α, and C1q, thereby activating astrocytes. These reactive astrocytes subsequently release neurotoxins, including lipocalin-2 (LCN2), which contribute to neurotoxicity. LCN2 can also promote neuronal cell death when neurons are damaged due to aging-induced dysregulation of proteostasis. Activated microglia are also capable of secreting neurotoxins.

LCN2 is a small secretory glycoprotein with a molecular mass of 24–25 kDa. It is also known as neutrophil gelatinase-associated lipocalin (NGAL) or 24p3^77,78^. It binds to an iron (Fe) carrier known as a siderophore. LCN2 is secreted from various cells and can be detected in blood plasma and urine, making it an important biomarker of inflammation, infection, and organ damage^79,80^. In fact, *Lcn2* expression is upregulated and LCN2 secretion is increased after ischemic renal injury, making it detectable in urine^81^. When LCN2 is secreted, it binds to siderophores and sequesters iron. Following its ability to bind bacterial or fungal siderophores, secreted LCN2 can inhibit their iron utilization for growth, resulting in efficient performance as a bactericide or fungicide^78,82^. Specifically, in LCN2-deficient mice, the loss of iron sequestration ability leads to vulnerability to bacterial invasion, such as *Escherichia coli*^83^. LCN2 is widely expressed at low levels in various tissues; however, its concentration dramatically increases after injury, infection, or inflammatory stress, making it an acute-phase protein^20,84–86^. Following liver injury, LCN2 production has been shown to increase and play a protective role in the damaged liver^87^. In the case of brain injury with intracerebral hemorrhage, the release of iron may trigger the secretion of LCN2 to sequester the released iron and maintain iron homeostasis^88^. Under metabolic inflammatory stress conditions, such as obesity, increased LCN2 secretion is associated with the induction of proinflammatory cytokines^89^. Although LCN2 can have both positive and negative roles, depending on the microenvironment, this review focuses on its potential adverse effects on the CNS. When LCN2 is overexpressed or a recombinant LCN2 protein is supplied to astrocytes, it leads to astrocyte activation, upregulation of GFAP, and morphological changes^90^. The neurotoxic or neuroprotective nature of LCN2 secreted from reactive astrocytes in the CNS is controversial, with conflicting evidence, possibly attributable to differences in the neuronal microenvironment or experimental protocols^22,66,91,92^. However, further evidence supports a neurotoxic role of LCN2 in the CNS^93^. LCN2 also plays a crucial role in lipopolysaccharide (LPS)-induced neuroinflammation and neurotoxicity^94,95^ (Fig. 2). Despite the expectation that activated astrocytes after ischemic brain injury are predominantly of the neuroprotective A2 subtype, with low *Lcn2* expression levels, it has been reported that LCN2 levels increase even under these conditions. Interestingly, LCN2 deficiency attenuates neuroinflammation and neuronal cell death after brain injury or CNS diseases^96,97^. Considering that *Lcn2* expression levels are increased in the neurotoxic A1 subtype, these findings strongly suggest that LCN2 is involved in neuronal apoptosis.

## The potential of LCN2 as a biomarker of neurodegenerative diseases

**Fig. 3:**
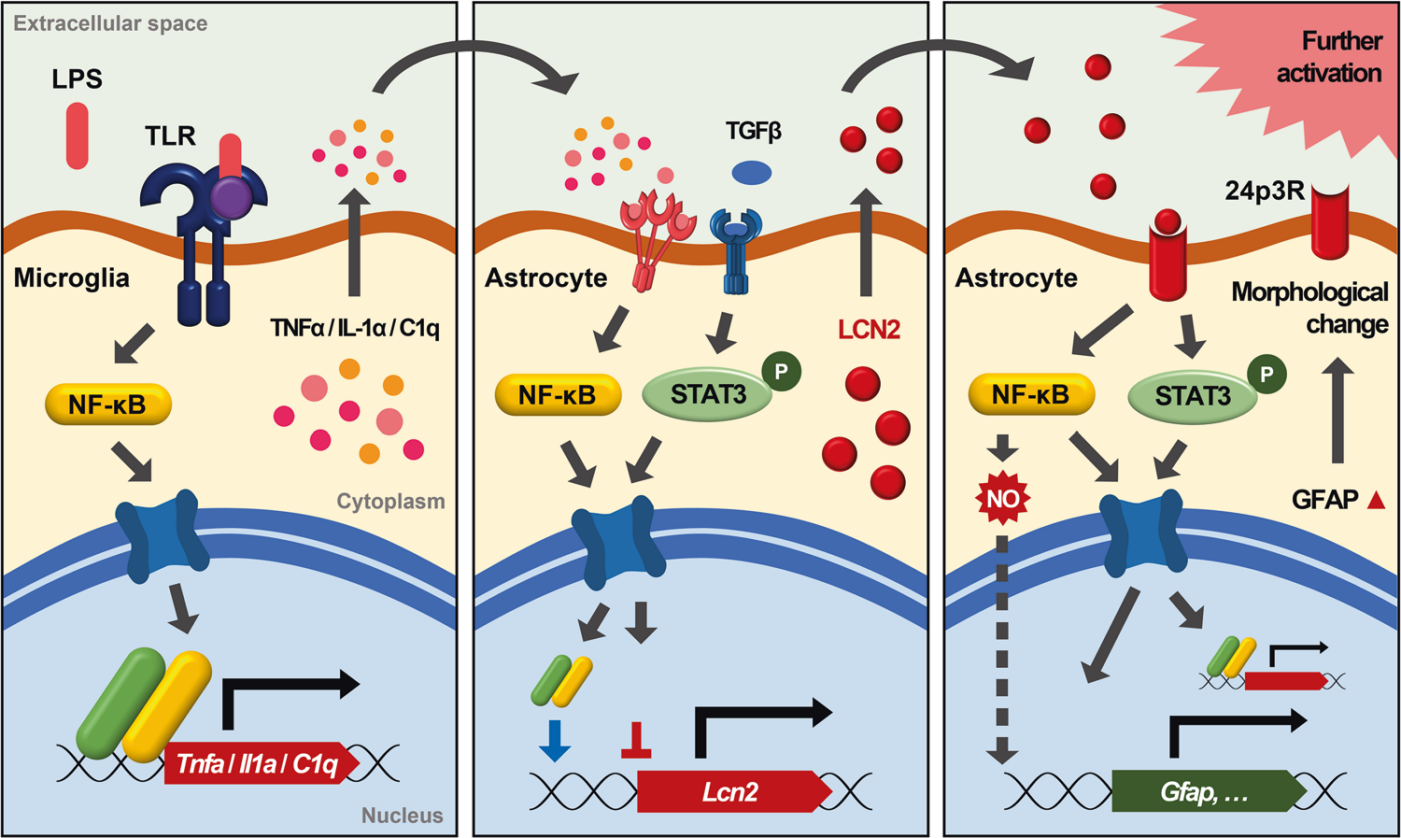
LCN2 is a key protein that induces and maintains astrocyte activation. LPS selectively binds to Toll-like receptor (TLR) 4 in microglia, triggering the activation of NF-κB signaling and the subsequent production of proinflammatory cytokines, such as TNFα, IL-1α, and C1q. These proinflammatory cytokines then activate astrocytes and induce the secretion of LCN2. It is well-established that anti-inflammatory cytokines, such as TGFβ, inhibit the production of LCN2. LCN2, in turn, binds to its receptor 24p3R in an autocrine or paracrine manner, promoting the activation of the NF-κB signaling pathway and further influencing the activation status of astrocytes. In addition, activation of the JAK/STAT3 signaling pathway and nitric oxide (NO) generated from the NF-κB signaling pathway increase *Gfap* expression levels, resulting in morphological changes in activated astrocytes.

The activation of glial cells by inflammatory stress, such as LPS treatment, is very similar to the activation of glial cells observed in neurodegenerative diseases. In the CNS, LPS administration induces the upregulation of *Lcn2* expression through the activation of the NF-κB signaling pathway^15,16,21^. Microglia, the resident immune cells in the brain, are activated by signals from damaged neurons or inflammatory stress^98,99^. Activated microglia increase LCN2 secretion, which may be autoregulatory, making them susceptible to NO-induced apoptosis^98^. In addition, activated microglia can activate astrocytes by secreting proinflammatory cytokines. Therefore, communication between microglia and astrocytes is required for LPS-induced reactive astrogliosis^13^ (Fig. 2). Microglia respond preferentially to inflammatory stress induced by LPS because they are more sensitive to pathogens and have higher expression levels of Toll-like receptor (TLR) 4 than astrocytes^100,101^. When proinflammatory cytokines, such as TNFα, IL-1α, and C1q, secreted through the activation of TLR signaling in microglia bind to cytokine receptors in astrocytes, *Lcn2* expression is upregulated through the activation of NF-κB signaling^71^ (Fig. 3). NF-κB serves as a master regulator that induces an increase in *Lcn2* expression, and NF-κB signaling activity is much higher in glial cells than in neurons. LCN2 secreted from activated astrocytes binds to the LCN2 receptor 24p3R in astrocytes and causes morphological changes (Fig. 3). Furthermore, it leads to neuronal cell death and additional activation of astrocytes and microglia. Thus, under neuroinflammatory stress conditions, proinflammatory cytokines released from microglia induce alterations in the transcriptome profiles of quiescent astrocytes, transforming them into reactive astrocytes with high expression levels of A1 markers and the secretion of neurotoxins, such as LCN2^13^. Consequently, astrocytes play a more important role in neuronal cell death than microglia.

In fact, TNFα, IL-1α, and C1q secreted from activated microglia are necessary and sufficient to activate astrocytes into the A1 subtype^13^. Neither activated microglia nor LPS are required if these proinflammatory cytokines are provided to astrocytes. In addition, it has been reported that the A1 subtype can be converted back to nonactivated or quiescent astrocytes through treatment with the anti-inflammatory cytokine TGFβ or fibroblast growth factor (FGF)^13^. These results support the possibility of A1-A2 conversion, in which transcriptome profiles change with decreased A1 marker expression levels and increased A2 marker expression levels. As secreted LCN2 further promotes the production of proinflammatory cytokines in response to inflammatory stress, it serves as a diagnostic marker for neuroinflammation, potential neuronal cell death, and the induction of brain damage. Therefore, LCN2 secreted from activated astrocytes is a promising biomarker for the early prediction, diagnosis, and inhibition of the progression of neurodegenerative diseases.

## Astrocyte activation and the regulation of LCN2

**Fig. 4:**
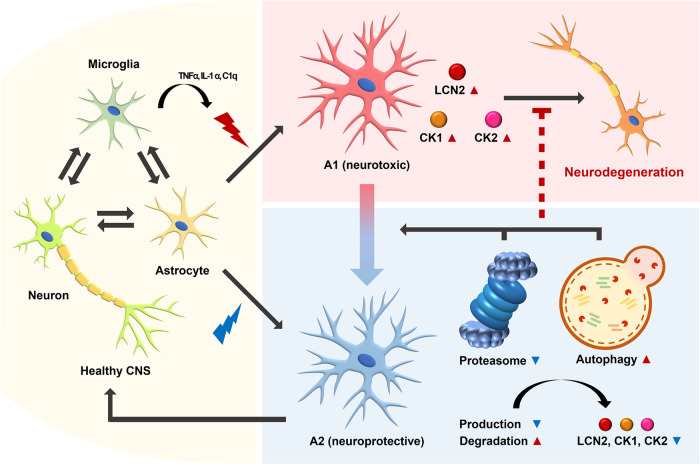
Effect of reducing LCN2 levels. Quiescent astrocytes can be activated into neurotoxic A1 subtypes by proinflammatory cytokines, such as TNFα, IL-1α, and C1q. However, the levels of LCN2 (and potentially other proinflammatory cytokines, such as CK1 and CK2) in activated astrocytes can be reduced by proteasome inhibition or autophagy activation. Proteasome inhibition suppresses the production of LCN2, and autophagy activation promotes the degradation of LCN2. This reduction in LCN2 levels may lead to the conversion of astrocytes into neuroprotective A2 subtypes.

LCN2 is regulated by NF-κB signaling at the transcriptional level but can be directly or indirectly downregulated by miRNAs after transcription^102,103^. Studies on the regulation of LCN2 at the protein level have mainly focused on inhibiting LCN2 secretion, inhibiting the action of secreted LCN2 using neutralizing antibodies, or using antagonists that block the binding of LCN2 to its receptors. Recently, it was shown that LCN2 is also regulated at the posttranslational level^22^. It is targeted to the autophagy‒lysosome pathway for degradation, likely to regulate the levels of secreted LCN2. LCN2 also affects lysosomal function and reduces autophagic flux in cardiac muscle cells, suggesting that LCN2 is not degraded by the autophagy‒lysosome pathway in these cells^104^. It is expected that de novo-produced LCN2 and endocytosed LCN2 reside in different intracellular compartments and interact with the autophagic machinery in different ways. Alternatively, the relationship between LCN2 and autophagy may be cell-type-dependent. The suppression of NF-κB signaling by inhibiting the degradation of IκB through proteasome inhibition reduces *Lcn2* expression levels even under inflammatory stress conditions. Furthermore, autophagy activation promotes the degradation of LCN2. Both proteasome inhibition and autophagy activation have been proven to be effective methods for reducing the secretion of LCN2 and alleviating neurotoxicity (Fig. 4).

LCN2 secreted from activated astrocytes binds to the LCN2 receptor 24p3R in an autocrine manner, further activating astrocytes. It can also bind to the LCN2 receptors of other cells, including inactive astrocytes, microglia, or neurons, in a paracrine manner. This chain reaction promotes an inflammatory response in the brain. Cell surface expression of the LCN2 receptor is generally higher in neurons than in astrocytes, making neurons more susceptible to the LCN2 response^21^. LCN2 bound to its receptor can be endocytosed, which affects intracellular iron homeostasis^105^. When LCN2 is loaded with iron, the intracellular iron concentration increases. In contrast, if LCN2 lacks iron, it may bind to the iron-siderophore complex and be removed by exocytosis, decreasing the intracellular iron concentration, disrupting iron homeostasis, and resulting in apoptosis^24^. In other cell types, such as cardiomyocytes, an increase in the intracellular iron concentration induces apoptosis^106^. Thus, LCN2-induced disruption of iron homeostasis may have different outcomes depending on the cell type. When LCN2 binds to the LCN2 receptor in astrocytes and activates NF-κB signaling, rather than JAK/STAT3 signaling, it is considered to be activated into the A1 subtype. Considering that the LCN2 level is high in the A1 subtype and low in the A2 subtype, there may be a correlation between the LCN2 level and the signaling process that is promoted or activated. Thus, LCN2 level-dependent selection of the NF-κB or JAK/STAT3 signaling pathways may be possible.

The LCN2 protein is found in neutrophil granules and is known to form a complex with the gelatinase MMP-9^19^. The LCN2-MMP-9 complex inhibits MMP-9 degradation. However, when LCN2 is polyaminated or deamidated, the formation of the complex is attenuated and the degradation of MMP-9 is promoted^107^. However, the effect of complex formation on the activity and stability of LCN2 is not well understood. LCN2 is rapidly degraded by the autophagy‒lysosome pathway inside cells, with a half-life of approximately 30 min^22^. Furthermore, this degradation is dependent on the presence of an N-terminal signal peptide (SP) that targets LCN2 to the endoplasmic reticulum for N-glycosylation^18,108^. The N-terminal SP is subsequently removed, and LCN2 is directed toward either the degradation or the secretory pathway. In fact, the N-terminal SP is required for both the degradation of LCN2 by autophagy and its secretion outside of cells^22^. Removal of the N-terminal SP is necessary before degradation or secretion of LCN2 can occur. Further research is required to fully understand the regulation of LCN2 degradation and secretion.

## Ubiquitin deficiency and reactive astrogliosis

It has been suggested that astrocytes, when activated by damaged neurons, secrete LCN2, which then promotes neuronal cell death^66^. The death of neurons with polyubiquitin gene *Ubb* KO may also be attributed to LCN2 secreted by activated astrocytes. Recently, in an animal model with activated astrocytes, it was discovered that H_2_O_2_ secreted by these astrocytes induces neuronal cell death^109^. Pharmacological interventions targeting reactive astrocytes are also effective at alleviating the pathogenesis of neurodegenerative diseases^110^.

In beta-amyloid precursor protein (APP)-deficient mice (B6.129S7-Apptm1Dbo/J), astrocyte activation occurs at 3 months, without significant signs of neuronal apoptosis^9^. Conversely, in *Ubb* KO mice, neuronal apoptosis occurs at 3 months after astrocyte activation in the hypothalamus of 1-month-old mice^10,11^. Thus, there is sufficient time to alter the neuronal microenvironment through astrocyte subtype conversion, even after astrocyte activation into the A1 subtype. Because mammals have two paralogous polyubiquitin genes, *Ubb* and *Ubc*, *Ubb* KO typically results in the upregulation or compensatory expression of the other polyubiquitin gene, *Ubc*. However, in certain brain regions, such as the arcuate nucleus of the hypothalamus, *Ubb* KO mice exhibit reduced levels of free ubiquitin (Ub). This is likely due to the low basal *Ubc* expression level in this brain region, resulting in insufficient compensatory expression to fully restore free Ub levels. Decreased levels of free Ub are closely associated with the onset and progression of neurodegenerative diseases, astrocyte activation, and neuronal cell death. Under certain circumstances, an increase in the formation of ubiquitinated protein aggregates or impaired protein degradation may contribute to decreased free Ub levels. Therefore, the relationship between decreased free Ub levels and neurodegenerative diseases remains a subject of debate, as it is unclear whether this decrease is a cause or a consequence of the disease^111–114^.

Free Ub refers to Ub monomers that are not conjugated to substrates or enzymes and are readily available. Aggregate formation leads to reduced levels of free Ub, resulting in decreased proteasome activity and compromised cell viability^115^. Conversely, a decrease in free Ub levels causes aggregate accumulation and impairs cell viability by reducing autophagy activity^116^. Even in the absence of aggregate formation, a decrease in free Ub levels leads to decreased proteasome activity and impaired cell proliferation^117^. Free Ub serves as a biomarker for the diagnosis and monitoring of the progression of neurodegenerative diseases. Therefore, the level of free Ub plays a crucial role in determining neurodegeneration or neuroprotection. During brain aging or neurodegenerative diseases, the level of free Ub in brain cells is reduced.

Ub deficiency also appears to upregulate *Lcn2* expression in mixed neuronal cells^118^. This may be due to a higher percentage of GFAP-positive astrocytes in *Ubb* KO or knockdown (KD) cells than in wild-type cells. Therefore, it is important to determine whether *Lcn2* expression is upregulated in *Ubb* KO or KD astrocytes under normal or neuroinflammatory stress conditions. It would also be interesting to investigate whether *Ubb* KO astrocytes exhibit increased expression levels of A1 markers.

## Conclusions and future perspectives

Neuronal cell death observed in neurodegenerative diseases may not solely be attributed to the neurons themselves. A different perspective is needed, considering that it may be caused by neurotoxins, such as LCN2, which are secreted from activated astrocytes exhibiting the A1 subtype. Therefore, future research should focus on proposing strategies to reduce LCN2 levels and convert activated astrocytes into the neuroprotective A2 subtype capable of safeguarding neuronal cells. This novel approach addresses the challenges posed by neurodegenerative diseases that have proved difficult to resolve through long-standing research focusing solely on neurons. LCN2 plays a pivotal role in initiating and sustaining astrocyte activation. The secretion of LCN2 from activated astrocytes influences the activation of neighboring astrocytes in both autocrine and paracrine manners. Although antibody treatment may be an effective method to block the function of LCN2 and the LCN2 receptor signaling pathway, there are also two alternative approaches that can effectively reduce LCN2 by blocking its transcriptional upregulation and accelerating its degradation. Both methods are applicable to all types of cells, including astrocytes involved in the production of LCN2 in the CNS, by utilizing the common feature of the intracellular degradation system. By reducing the level of LCN2, it is possible to convert neurotoxic A1 subtype astrocytes into the neuroprotective A2 subtype. This approach offers a more effective means of preventing neuronal cell death than inhibiting astrocyte activation, which is challenging under aging or neuroinflammatory stress conditions.
